# Identification of ribosomal protein family in triple-negative breast cancer by bioinformatics analysis

**DOI:** 10.1042/BSR20200869

**Published:** 2021-01-06

**Authors:** Ziyue Lin, Rui Peng, Yan Sun, Luyu Zhang, Zheng Zhang

**Affiliations:** 1 Department of Molecular Medicine and Cancer Research Center, Chongqing Medical University, Chongqing, China; 2 Department of Bioinformatics, Chongqing Medical University, Chongqing, China

**Keywords:** bioinformatics analysis, DEGs, non-TNBC, survival analysis, TNBC

## Abstract

Triple-negative breast cancer (TNBC) accounts for ∼20% of all breast cancer (BC) cases. The management of TNBC represents a challenge due to its worse prognosis, heterogeneity and lack of targeted therapy. Moreover, its mechanisms are not fully clear. The aim of the study is to identify crucial genes between TNBC and non-TNBC for underlying targets for diagnostic and therapeutic methods of TNBC. The differentially expressed genes (DEGs) between TNBC and non-TNBC were selected from the Gene Expression Omnibus (GEO) database after the integrated analysis of two datasets (GSE65194 and GSE76124). Then Gene ontology (GO) and KEGG analysis were performed by DAVID database, protein–protein interaction (PPI) of DEGs was constructed by Search Tool for the Retrieval of Reciprocity Genes (STRING) database. Furthermore, centrality analysis and module analysis were carried out by Cytoscape to analyze the TNBC-related PPI. Subsequently, overall survival (OS) analysis was performed by GEPIA. Finally, the expressions of these key genes in TNBC and non-TNBC tissues were tested by qRT-PCR. The results showed that 955 DEGs were obtained, which were mainly enriched in ribosome, ribosomal subunit, and so on. Moreover, 19 candidate genes were focused on by centrality analysis and module analysis. Furthermore, we found the low expressions of ribosomal protein S9 (*RPS9*), ribosomal protein S14 (*RPS14*), ribosomal protein S27 (*RPS27*), ribosomal protein L11 (*RPL11*) and ribosomal protein L14 (*RPL14*) were related to a poor OS in BC patients. Additionally, qRT-PCR results suggested that these five genes were notably down-regulated in TNBC tissues. In summary, the present study suggests that ribosomal proteins are related to TNBC, and they may play an important role in the diagnosis, treatment and prognosis of TNBC.

## Introduction

Breast cancer (BC) is the most common malignant disease worldwide and remains a major health problem among women [1]. Triple-negative BC (TNBC) is a particular subtype of BC, which is diagnosed by lacking the expression of estrogen receptor (ER), progesterone receptor (PR), and human epidermal growth factor receptor 2 (HER2), and accounts for approximately 15–20% of all BC [2]. Molecular expression profile, reflecting clinical heterogeneity, has set up five types of breast carcinomas that carry different properties of prognoses and survivals: Luminal A, Luminal B, Her2(+), normal and triple negative subtypes [3]. TNBC typically behaves more aggressively, worse overall survival (OS) and requires more complicated treatment approaches compared with non-TNBC [4]. Correct early diagnosis assessment of BC is very difficult, even though many cancer-related genes and cellular pathways related to BC have appeared [5]. The common treatments of BC are surgery, chemotherapy, radiotherapy and endocrine therapy, such as endocrine therapy for hormone receptor-positive patients and trastuzumab for HER2-positive patients, but there are no valid treatment tactics available to treat TNBC [6]. TNBC patients frequently suffer higher risks of distant recurrence and distal metastasis, and higher possibility of relapse, causing poor prognosis [7]. Therefore, it is vitally important to explore potential diagnostic and prognostic biomarkers and therapeutic targets of TNBC.

The fast development of gene microarray technology and bioinformatics analysis based on high-throughput data, provide new tactics to identify differentially expressed genes (DEGs) and discover therapeutic targets for the initiation and evolution of BC [8]. Aberrant expression of genes plays an important role in the initiation and progression of tumors, so mastering the alteration in the characteristics of critical genes promotes to comprehensively understand TNBC progression and screen related molecular markers [9]. Recently, studies identified low expression of ITSN1 in BC tissues and cell lines by bioinformatics analysis [5]. Indeed, some researchers found key genes and pathways in TNBC by integrated bioinformatics analysis between TNBC and non-TNBC [10]. However, the use of bioinformatics analysis method to find the relevant genes of TNBC has not yet been confirmed.

In the current study, we aimed to gain the crucial genes between TNBC and non-TNBC. We identified 955 DEGs by comparing the genes expression profiles of samples from TNBC and non-TNBC patients and constructed the TNBC-related protein–protein interaction (PPI) network. Moreover, gene ontology (GO) showed the DEGs enriched in ribosome, ribosomal subunit, cytosolic ribosome, structural constituent of ribosome, RAGE receptor binding. And Kyoto Encyclopedia of Genes and Genomes pathway (KEGG) analysis displayed that the significant pathways included ribosome, FOXO signaling pathway, HIF-1 signaling pathway. Further, 19 candidate genes with high centrality values and located at the first module were found by the centrality analysis and module screening in the TNBC-related PPI network. Interesting, the results of survival analysis displayed ribosomal protein family genes were related to a poor OS among these candidate genes, including ribosomal protein S9 (RPS9), ribosomal protein S14 (RPS14), ribosomal protein S27 (RPS27), ribosomal protein L11 (RPL11) and ribosomal protein L14 (RPL14). Additionally, quantitative real-time PCR (qRT-PCR) results showed the expressions of RPS9, RPS14, RPS27, RPL11 and RPL14 were notably down-regulated in tumor tissues of 16 TNBC patients when compared with those in 21 cases of non-TNBC patients. The present study might provide further insight into the ribosomal proteins for prognosis and drug discovery in TNBC.

## Materials and methods

### Access to public data

Two expression profiling datasets (GSE65194 and GSE76124) were downloaded from the Gene Expression Omnibus (GEO) database (https://www.ncbi.nlm.nih.gov/geo/), an open platform for storing genetic data. Their screening criteria are as follows: (a) they were updated recently (2019); (b) the sample size of each dataset would be greater than 150; (c) the samples are from the same platform: GPL570 [HG-U133_Plus_2] Affymetrix Human Genome U133 Plus 2.0 Array. The GSE65194 dataset contained 178 arrays. A total of 153 arrays were used to analyze 130 unique BC samples (41 cases of TNBC; 89 cases of non-TNBC) and 23 technical duplicates. In addition 11 normal breast tissue samples and 14 TNBC cell lines were included. GSE76124 consisted of 198 TNBC tumors (discovery set: *n*=84; validation set: *n*=114).

### Screening for DEGs

In order to find out DEGs between in TNBC and non-TNBC breast tissues in the GSE76124 and GSE65194 datasets, we first removed the normal samples and cell lines, and then left TNBC and non-TNBC cancer samples. Through R affymetrix package analysis, we obtained the list of DEGs of the two microarray datasets. The list of down-regulated and up-regulated genes in the microarray data were saved. *P*<0.05 and |fold change| > 1.5 were used as the cut-off criteria of DEGs.

### GO and KEGG pathway analysis

GO is a bioinformatics tool and used to annotate genes and analyze biological functions of DEGs or key genes. In addition, GO offers three categories of defined terms, which include biological processes (BP), cellular component (CC), and molecular function (MF). KEGG, as a sophisticated database resource for the systematic analysis of gene functions, links genomic information and high-order functional information. DAVID [11] (https://david.ncifcrf.gov/) was used to perform the GO and KEGG analysis. The species was limited to *Homo sapiens* and *P*<0.05 was set as the cut-off criterion.

### Construction of PPI network

The Search Tool for the Retrieval of Reciprocity Genes (STRING) database [11] (http://string-db.org/), which collects and predicts interaction information from genomic context predictions, high-throughput lab experiments, co-expression, automated text-mining and previous knowledge from databases, was used to predict the potential interactions between gene candidates at the protein level. The combined score of medium confidence > 0.4 was used as the cut-off value in the STRING database. In the PPI network, certain DEGs on the margins and isolated, suggesting no association with other genes, were removed.

### Centrality analysis of PPI network

Centrality analysis which is used to identify the vital nodes in networks is of great significance for understanding the function of nodes and the nature of networks [12]. Many centrality indices have been proposed to identify the influential nodes of networks. Typically, we predicted the key genes of the network using the significant graph-theoretic measures of degree centrality, betweenness centrality and closeness centrality [13]. The scores of three centralities (degree centrality, closeness centrality and betweenness centrality) were calculated by cytoNCA, a plugin of Cytoscape for network centrality analysis. Then we used R language to describe the distribution of the three parameters and calculate the correlation among the three key centralities. Finally, the Venn diagram presented the intersections of the top 30% degree value, top 30% betweenness value and top 30% closeness value.

### Module analysis of PPI network

MCODE, a plug-in Cytoscape, is applied to screen the modules considered to be the essential part of the network [14]. And degree cut-off = 2, node density cut-off = 0.1, node score cut-off = 0.2, k-core = 2 and max depth = 100 were regarded as the criteria. The genes in the first ranked Module with high degree, betweenness and closeness values were selected as candidate genes for further analysis.

### Survival analysis of Hub genes

The OS analysis of 19 candidate genes was performed using the online GEPIA survival analysis server (http://gepia.cancer-pku.cn/) [15], which included 9736 tumor tissues and 8587 normal tissues. The *P*<0.05 was considered to indicate statistical significance, and screened out promising hub genes with the prognostic value.

### Tissue samples

The samples of 16 TNBC tissues and 21 non-TNBC tissues were collected from the First Affiliated Hospital of Chongqing Medical University (Chongqing, China) without chemotherapy or radiotherapy before surgical excision and all patients gave informed consents. Tissue samples were snap-frozen in liquid nitrogen for further analysis. All samples specimens from the patients were diagnosed with TNBC or non-TNBC by biopsy specimen immunohistochemistry staining. The study has been approved by the Ethics Committee of the Chongqing Medical University, and was conducted in compliance with the Helsinki Declaration.

### RNA isolation and determination of target gene expression using qRT-PCR

To confirm our bioinformatics results, qRT-PCR were conducted on TNBC and non-TNBC tissues. Total RNA was prepared using TRIzol reagent (TaKaRa, China). The isolated RNA was reverse-transcribed into cDNA using a reverse transcription kit (TaKaRa, China). The qRT-PCR procedure was performed was as follows: 3 min for 95°C, 40 cycles at (95°C for 15 s, 60°C for 30 s, 72°C for 30 s), 65°C for 5 s, 95°C for 50 s. Each qRT-PCR was performed in triplicate on samples. The primers used for the validation are listed in Table 1. The relative quantitative data of mRNAs were normalized to GAPDH and quantified using the 2^−ΔΔ*C*_t_^ method (Δ*C*_t_ = *C*_t_ target gene − *C*_t_ internal control) [16].

**Table 1:** The primer sequence of the five key genes

Gene symbol	Primer category	Primer sequence
*RPS9*	Forward primer	GAAATCTCGTCTCGACCAAGAG
	Reverse primer	GGTCCTTCTCATCAAGCGTCA
*RPS14*	Forward primer	CCATGTCACTGATCTTTCTGGC
	Reverse primer	TCATCTCGGTCTGCCTTTACC
*RPS27*	Forward primer	ATGCCTCTCGCAAAGGATCTC
	Reverse primer	TGAAGTAGGAATTGGGGCTCT
*RPL11*	Forward primer	AAAGGTGCGGGAGTATGAGTT
	Reverse primer	TCCAGGCCGTAGATACCAATG
*RPL14*	Forward primer	GACCTTGCACTCAAGTGAGGA
	Reverse primer	CTTGTCGGACATACTTCTGGTG
*GAPDH*	Forward primer	ACAACTTTGGTATCGTGGAAGG
	Reverse primer	GCCATCACGCCACAGTTTC

### Statistical analysis

Statistical analyses were performed in GraphPad Prism 5.0 software. The values of different groups were represented by the mean ± SD. The comparison of expression levels of TNBC and non-TNBC tumor tissues were analyzed by unpaired *t* test. *P*<0.05 was considered to represent a statistically significant difference.

## Results

### Identification of DEGs

Generally, 239 patients with TNBC and 89 patients with non-TNBC were incorporated into the present study by integrating and screening samples of GSE65194 and GSE76124. The fundamental characteristics of the TNBC and non-TNBC were summarized in Table 2. The results displayed TNBC was positively correlated with body mass index (*P*<0.0001), tumor size (*P*<0.0001) and metastases (*P*<0.001), but not with other clinical pathological parameters including age and menopausal status.

**Table 2:** Clinical characteristics of the TNBC and non-TNBC patients

Characteristic	All tumors		TNBC		Non-TNBC		Chi-square	*P*-value
	*n*	%	*n*	%	*n*	%		
**Age (years)**	322		233		89			
<50	126	39.13	93	39.91	33	37.08	0.217	0.641
≥50	196	60.87	140	60.09	56	62.92		
Missing	6		6		0			
**Menopausal status**	297		208		89			
Premenopausal	129	43.43	88	42.31	41	46.07	0.359	0.549
Postmenopausal	168	56.57	120	57.69	48	53.93		
Missing	31		31		0			
**Body mass index**	294		206		88			
Underweight (<18.5)	7	2.38	3	1.46	4	4.55	22.313	0.000
Normal (18.5–24.9)	115	39.12	65	31.55	50	56.82		
Overweight (25–29.9)	94	31.97	72	34.95	22	25.00		
Obese (≥30)	78	26.53	66	32.04	12	13.64		
Missing	34		33		1			
**Tumor size (cm)**	312		232		80			
<2	81	25.96	44	18.97	37	46.25	23.052	0.000
2–5	203	65.06	165	71.12	38	47.50		
>5	28	8.97	23	9.91	5	6.25		
Missing	16		7		9			
**Metastases**	276		187		89			
No metastases	246	89.13	173	92.51	73	82.02	6.85	0.009
Metastases found	30	10.87	14	7.49	16	17.98		
Unknown	52		52		0			

**Figure 1:**
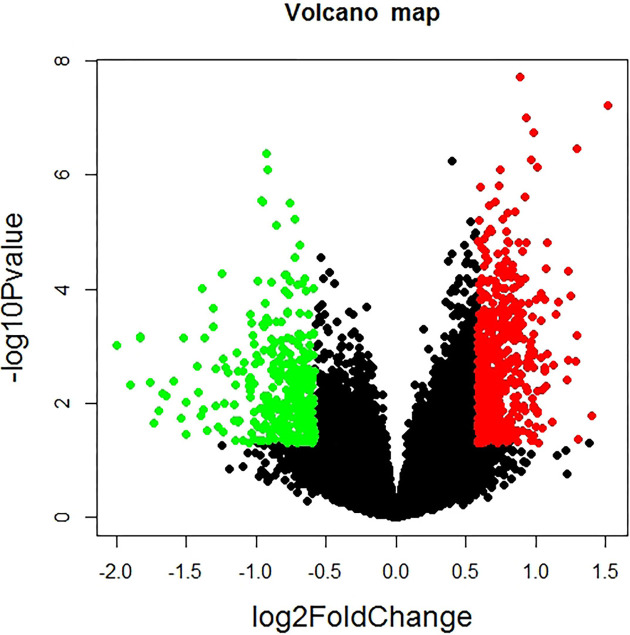
Volcano plot demonstrating the differential expression of all genes Red points mean up-regulated genes screened on the basis |log2 fold change| ≥ 0.585 and *P*-value <0.05. Green points mean down-regulated genes screened on the basis |log2 fold change| ≥ 0.585 and *P*-value <0.05. Black points represent genes with no difference in expression.

Through R affymetrix package process, we integrated the two microarray datasets and obtained 23506 genes. Further study, we identified 955 DEGs between TNBC and non-TNBC groups on the basis of |fold change| > 1.5 and *P*-value <0.05. Among these DEGs, 587 genes were up-regulated and 368 genes were down-regulated (Figure 1).

### Enrichment function analysis of GO and KEGG pathways

**Figure 2:**
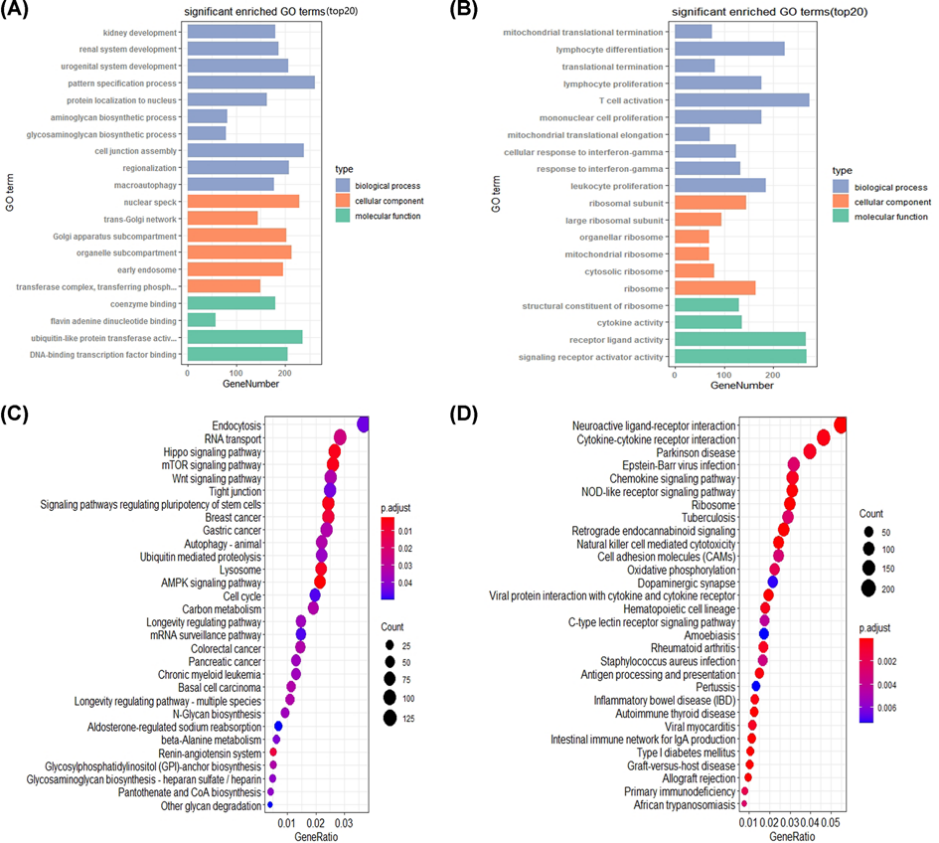
GO and KEGG enrichment analysis of the PPI network (**A**) Top 20 significantly enriched GO annotations of up-regulated DEGs, including three groups (molecular function, biological process and cellular component), *P*<0.05. (**B**) Top 20 significantly enriched GO annotations of down-regulated DEGs, *P*<0.05. (**C**) Top 20 functional pathways of up-regulated DEGs through KEGG analysis, *P*<0.05. (**D**) Top 20 functional pathways of down-regulated DEGs through KEGG analysis, *P*<0.05.

To gain in-depth and comprehensive biological characteristics of these DEGs, GO functional annotation and KEGG signaling pathway enrichment analysis were performed through online analytical tool DAVID. The results of the GO analysis demonstrated that DEGs significantly enriched in ‘pattern specification process’, ‘nuclear speck’, ‘ubiquitin like-protein transferase active’ (Figure 2A), ‘ribosome’, ‘ribosomal subunit’, ‘large ribosomal subunit’, ‘cytosolic ribosome’, ‘mitochondrial ribosome’ and ‘structural constituent of ribosome’ (Figure 2B). In addition, KEGG pathway enrichment analysis indicated that significant pathways of DEGs included ‘Hippo signaling pathway’, ‘mTOR signaling pathway’, ‘Wnt signaling pathway’ (Figure 2C), ‘Ribosome’ and ‘Neuroactive ligand-receptor interaction’ (Figure 2D). Both GO and KEGG results showed the DEGs were enriched in ribosomal related terms or pathways. These results suggest that ribosome-related genes may play an important role in the occurrence and development of TNBC.

### Construction of the TNBC-related PPI network

To explore the functions of these DEGs, the TNBC-related PPI network was constructed by an online analysis tool STRING. Each gene was assigned a degree representing the number of neighboring nodes in the network and average node degree was 10.3. A total of 841 nodes and 4338 edges were screened from the network, as shown in Supplementary Figure S1.

### Centrality analysis of the PPI network

**Figure 3:**
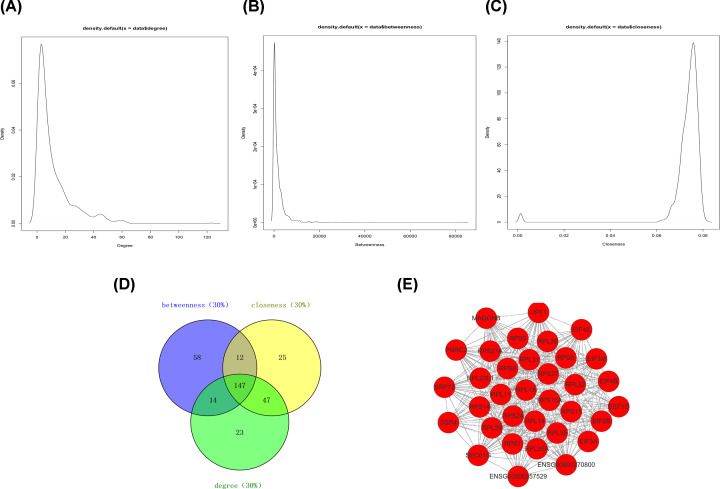
Centrality analysis and module analysis of the PPI network acquired through cytoNCA and MCODE plugins of Cytoscape (**A**) A density diagram of degree centrality. (**B**) A density diagram of betweenness centrality. (**C**) A density diagram of closeness centrality. (**D**) The intersection of the top 30% molecules in each centrality (degree, betweenness, closeness) was detected by Venny2.1. Results showed 147 key DEGs were chosen for further study because of their high degree, betweenness and closeness. (**E**) The most significant module was obtained from the PPI network which contained 34 nodes and 49 edges.

To study the features of the molecules in the TNBC-related PPI network, centrality analysis was performed using three topological parameters (degree, betweenness and centrality) in cytoNCA. Degree centrality is the most direct measure of the centrality of a node in network analysis. Betweenness centrality is an indicator of the importance of a node based on the number of shortest paths through a node. Closeness centrality reflects the proximity of a node to other nodes in the network. Our results showed that the density distributions of degree and betweenness displayed the long-tailed distribution [17], while closeness displayed fat-tailed distribution [18] (Figure 3A–C). The long-tailed distribution of degree and betweenness indicated that the parameter values of the vast majority of genes were small, and only a few genes had fairly large parameter values. The fat-tailed distribution of closeness indicated that the parameter values of the most genes were generally high. As known, in a PPI network, the greater the centrality value is, the more important the gene is. Therefore, 147 DEGs with high topological features (top 30% of each parameter) were obtained chosen for further study (Figure 3D).

### Module analysis of the PPI network

To further explore the characteristics of the molecules based on the PPI network, modules analysis was utilized by MCODE in Cytoscape software. The results showed 23 modules in the PPI network. The first-ranked module which was the most significant module was filtered from the PPI network, and it included 34 nodes and 499 edges with a highest score of 30.242 (Figure 3E). Ultimately, 19 candidate genes (*MAGOHB, RPL18, SSR4, EIF5B, SRP72, RPS6, RPS27A, UPF1, RPL32, RPS5, SEC61G, RPS9, RPL11, RPS27, EIF4B, RPL14, EIF4E, RPS14* and *RPL29*) in the network were focused on because of their high centrality values and the location of the first ranked module. Interestingly, 11 genes were ribosome-related genes among these 19 genes, pointing that ribosomal gene may play an important role in TNBC.

### OS analysis of candidate genes

**Figure 4:**
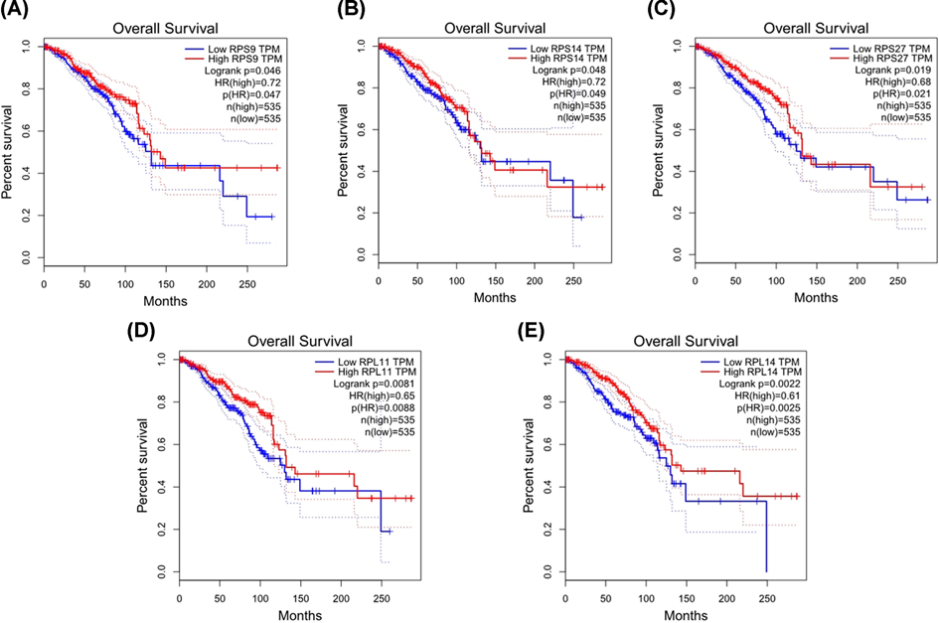
OS analysis of five key genes in BC patients using GEPIA (**A**) OS analysis of RPS9 in BC patients. The results showed the patients with low expression of RPS9 were related to a poor prognosis. (**B**) OS analysis of RPS14 in BC patients. The results showed the patients with low expression of RPS14 were related to a poor prognosis. (**C**) OS analysis of RPS27 in BC patients. The results showed the patients with low expression of RPS27 were related to a poor prognosis. (**D**) OS analysis of RPL11 in BC patients. The results showed the patients with low expression of RPL11 were related to a poor prognosis. (**E**) OS analysis of RPL14 in BC patients. The results showed the patients with low expression of RPL14 were related to a poor prognosis. *P*<0.05 was considered to indicate a statistically significant difference.

OS is often considered the best outcome endpoint in clinical trials of cancer. The prognostic value of 19 candidate genes in the PPI network was evaluated by using the online GEPIA survival analysis. OS for TNBC patients was obtained in the accordance with the low or high expression of the 19 candidate genes. Based on the screen criteria *P*<0.05, we obtained five key genes such as *RPS9* (*P*=0.047), *RPS14* (*P*=0.049), *RPS27* (*P*=0.021), *RPL11* (*P*=0.0088), *RPL14* (*P*=0.0025). As it is shown in Figure 4, a low expression of RPS9, RPS14, RPS27, RPL11 or RPL14 was associated with poor prognosis in the BC, suggesting they could help develop an interesting therapeutic approach against TNBC. Apparently, the results showed they all belonged to the ribosomal protein family, suggesting ribosome proteins may be closely related to the progress, prognosis and treatment of TNBC. Since these five key genes have the potential significance as prognosis biomarkers for TNBC, we selected them for further study.

### Validation of key genes by real-time qPCR

**Figure 5:**
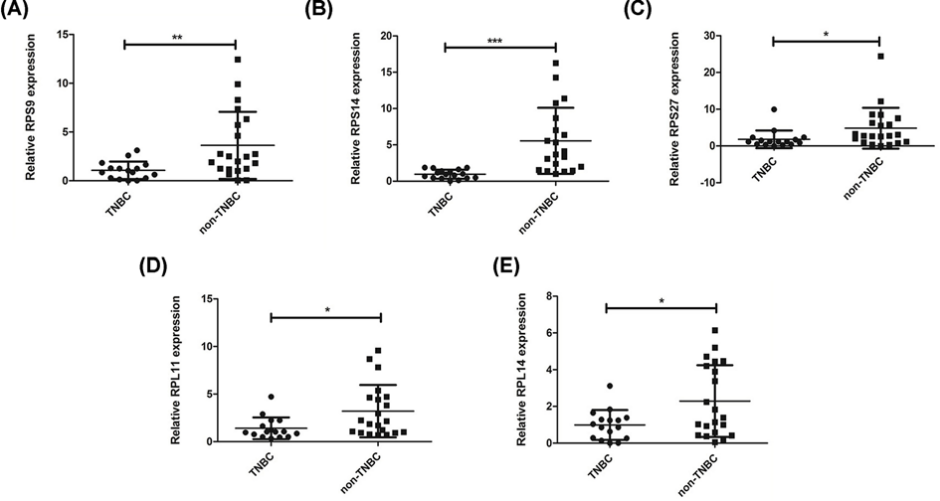
Validation of five key genes through qRT-PCR (**A**) The relative mRNA expression of RPS9 was lower in TNBC tissues than that in non-TNBC tissues. (**B**) The relative mRNA expression of RPS14 was lower in TNBC tissues than that in non-TNBC tissues. (**C**) The relative mRNA expression of RPS27 was lower in TNBC tissues than that in non-TNBC tissues. (**D**) The relative mRNA expression of RPL11 was lower in TNBC tissues than that in non-TNBC tissues. (**E**) The relative mRNA expression of RPL14 was lower in TNBC tissues than that in non-TNBC tissues (* means *P*<0.05, ** means *P*<0.01, *** means *P*<0.001).

To validate the key genes participating in the pathogenesis of TNBC, we performed qRT-PCR to detect the expression of five key genes in 16 TNBC tissues and 21 non-TNBC tissues. Our results showed the expressions of RPS9, RPS14, RPS27, RPL11 and RPL14 were significantly decreased in TNBC tissues when compared those in non-TNBC group (Figure 5). Moreover, the down-regulated expression results of these genes were verified to be consistent with the microarray results. These results suggest ribosomal proteins may participate in the development of TNBC and serve as the potential biomarker.

## Discussion

BC, the most common malignancy in women, exhibits significant heterogeneity [19]. Due to the absence of druggable molecular targets, the treatment of TNBC is very limited compared with the treatment of non-TNBC subtypes. However, the molecular mechanisms of TNBC remain poorly understood. The identification of biomarkers associated with TNBC tumorigenesis and progression are urgently required. Recently, microarray technology and bioinformatics analysis has enabled researchers to explore genetic alterations, and have been a useful approach to identify novel biomarkers in several diseases, such as adrenocortical carcinoma [20]. Additionally, high-throughput technology is increasingly advanced and widespread. Simultaneously, with the development of numerous public databases, like the GEO, transcriptomic and genomic research get great convenience from these public databases. Thus, based on these public platforms, we used bioinformatics analysis to explore the transcriptional differences between TNBC and non-TNBC to better understand BC and provide new clues of targeted therapy for TNBC.

In our study, by the analysis of two gene expression profiles of GSE65194 and GSE76124, we identified 955 DEGs in TNBC compared with non-TNBC (*P*<0.05, FC > 1.5), including 587 up-regulated genes and 368 down-regulated genes. Moreover, GO enrichment analysis revealed that DEGs significantly enriched in ribosome, ribosomal subunit and cytosolic ribosome. The KEGG pathway database contains information on systematic analysis of gene functions, linking genomics with functional information. Also, the result of KEGG showed that the genes were mainly enriched in ribosome and FOXO signaling pathway. This result arouses us enormous interest. Both GO terms and KEGG pathways displayed the ribosomal related, suggesting ribosomal related genes may play roles in TNBC. About ribosome, controlled changes in ribosome heterogeneity would up- or down-regulate particular genetic networks [21]. It has been reported that when ribosomal protein is deleted or reduced, the ribosome biogenesis process is blocked [22] and ribosome biogenesis has recently emerged as an effective target in cancer therapy [23,24]. For instance, RPL10a/uL1 is required for translation of mRNAs that promote cell survival, while genes that contribute to cell death are depleted from RPL10a/uL1 containing ribosomes, suggesting that tuning the levels of RPL10a/uL1 could shift the balance between cell survival and death [25]. Some researchers revealed a series of eukaryotic-specific antibiotics that are specific to cytosolic ribosome, uncovering promising cancer targets for human ribosomes [26]. Therefore, the data suggest that the identified DEGs which enriched in ribosomal terms or pathways may participant in the development of TNBC and contribute to TNBC treatment.

To explore the molecular mechanism of TNBC, we constructed the TNBC-related PPI network. We screened 841 nodes and 4338 edges from the network. Moreover, we performed centrality analysis and acquired 147 DEGs with high topological features. As known, centrality analysis was a criteria of screening hub genes to identify DEGs in lung cancer [27]. To deeply explore the molecular features of TNBC, we detected 23 functional modules by module analysis. Importantly, the first ranked module was found including 34 nodes and 499 edges. According to previous studies, modules analysis have been widely used to identify the key genes in many cancers such as renal carcinoma [9], TNBC [28] and gastric adenocarcinoma [29]. Thus, these suggest that the genes in the first-ranked module may be the significant molecules of the network. Comprehensively considering centrality analysis and module analysis, we found 19 candidate genes in the first-ranked module with high degree, betweenness and closeness. These candidate genes acted as important molecules in the network. Therefore, it suggests that these 19 candidate molecules may be the important molecules in the TNBC compared with non-TNBC.

Survival analysis is important indicators for assessing the prognosis of diseases, especially in cancer research. Our results indicated that TNBC patients with the low expression genes of RPS9, RPS14, RPS27, RPL11 or RPL14 were significantly worse in survival and prognosis of BC. Furthermore, real-time PCR was used to confirm the expression of these five genes in 16 TNBC samples and 21 non-TNBC samples. Our real-time PCR results showed that RPS9, RPS14, RPS27, RPL11 and RPL14 were all down-regulated in TNBC samples compared with non-TNBC samples. Our findings suggested that these five genes were the most important differently expressed genes between TNBC and non-TNBC. RPS9, RPS14, RPS27, RPL11 and RPL14 all belong to the ribosomal protein family. Combined with the results of GO and KEGG analysis, these results imply that ribosomal protein family genes may participate in TNBC.

It is well known, ribosomal protein family are the cornerstone of ribosome biogenesis and are involved in ribosome assembly. Indeed, ribosomal proteins are typically small (50–150 amino acid residues) and basic proteins with high isoelectric points [30]. Ribosomal proteins play seminal roles in the function and structure of ribosomes or in the initiation, elongation, or termination phases of protein translation. In our results, RPS9, RPS14 and RPS27 are components of the 40S subunit, while RPL11 and RPL14 are components of the 60S subunit. Studies had shown that RPS9 expression was associated with OS and extramedullary infiltration in myeloma [31]. RPS9 promoted osteosarcoma tumor growth by activating MAPK signaling pathway [32]. Based on bioinformatics analysis, RPS14 was identified as hub gene of bladder carcinoma [33]. Overexpression of RPS14 can inhibit Rb phosphorylation and induce cell cycle arrest and aging, which may be related to cancer treatment [34]. Also, low expression of RPS27 is associated with poor clinical prognosis in melanoma [35]. RPL11, affecting osteosarcoma malignant phenotype, may be a new prognostic markers of osteosarcoma survival [36]. Furthermore, deletion of RPL11 blocked p53 activation to induce colon cancer cell apoptosis [37]. In addition, RPL14 was a diagnostic markers of glioblastoma and could be used as therapeutic targets [38]. Moreover, researches have shown that RPL14 promoted cervical cancer cell migration, invasion and EMT, which were related to cervical cancer prognosis [39]. Eminently, previous researches had indicated that RPS9, RPS14, RPL11were down-regulated in BC and the low expression of RPS9, RPS14, RPL11 linked to worse OS in BC patients [40]. Therefore, these molecules may be used as potential effective candidates for early diagnosis or prognosis of TNBC. Importantly, research on ribosomal proteins may be a significant new direction for the diagnosis, prognosis and treatment of TNBC.

In future researches, we will further validate the reliable biomarkers for TNBC by more functional search and more samples. Our final attempts are to find the reliable biomarkers for clinical examination and point new direction of therapy.

## Conclusion

We showed that ribosomal protein family genes *RPS9, RPS14, RPS27, RPL11* and *RPL14* are key DEGs which are associated with poor outcome in TNBC compared with non-TNBC. These genes may be potential diagnostic and prognostic biomarker and to some extent be helpful for targeted therapy of TNBC.

## Supplementary Material

Supplementary Figure S1

## Abbreviations

BC: breast cancer
DAVID: Database for Annotation, Visualization and Integrated Discovery
DEG: differentially expressed gene
ER: estrogen receptor
GEO: Gene Expression Omnibus
GEPIA: Gene Expression Profiling Interactive Analysis
GO: Gene Ontology
KEGG: Kyoto Encyclopedia of Genes and Genomes
OS: overall survival
PPI: protein–protein interaction
PR: progesterone receptor
qPCR: quantitative PCR
qRT-PCR: quantitative real-time PCR
RPL11: ribosomal protein L11
RPL14: ribosomal protein L14
RPS9: ribosomal protein S9
RPS14: ribosomal protein S14
RPS27: ribosomal protein S27
STRING: Search Tool for the Retrieval of Reciprocity Genes
TNBC: triple-negative breast cancer
