# Supplementary figures and images for: Identification of ribosomal protein family in triple-negative breast cancer by bioinformatics analysis
Source: Biosci Rep. 2021 Jan 6;41(1):BSR20200869. doi: 10.1042/BSR20200869 (PMC7789804; doi:10.1042/BSR20200869)

### Supplementary Figure S1

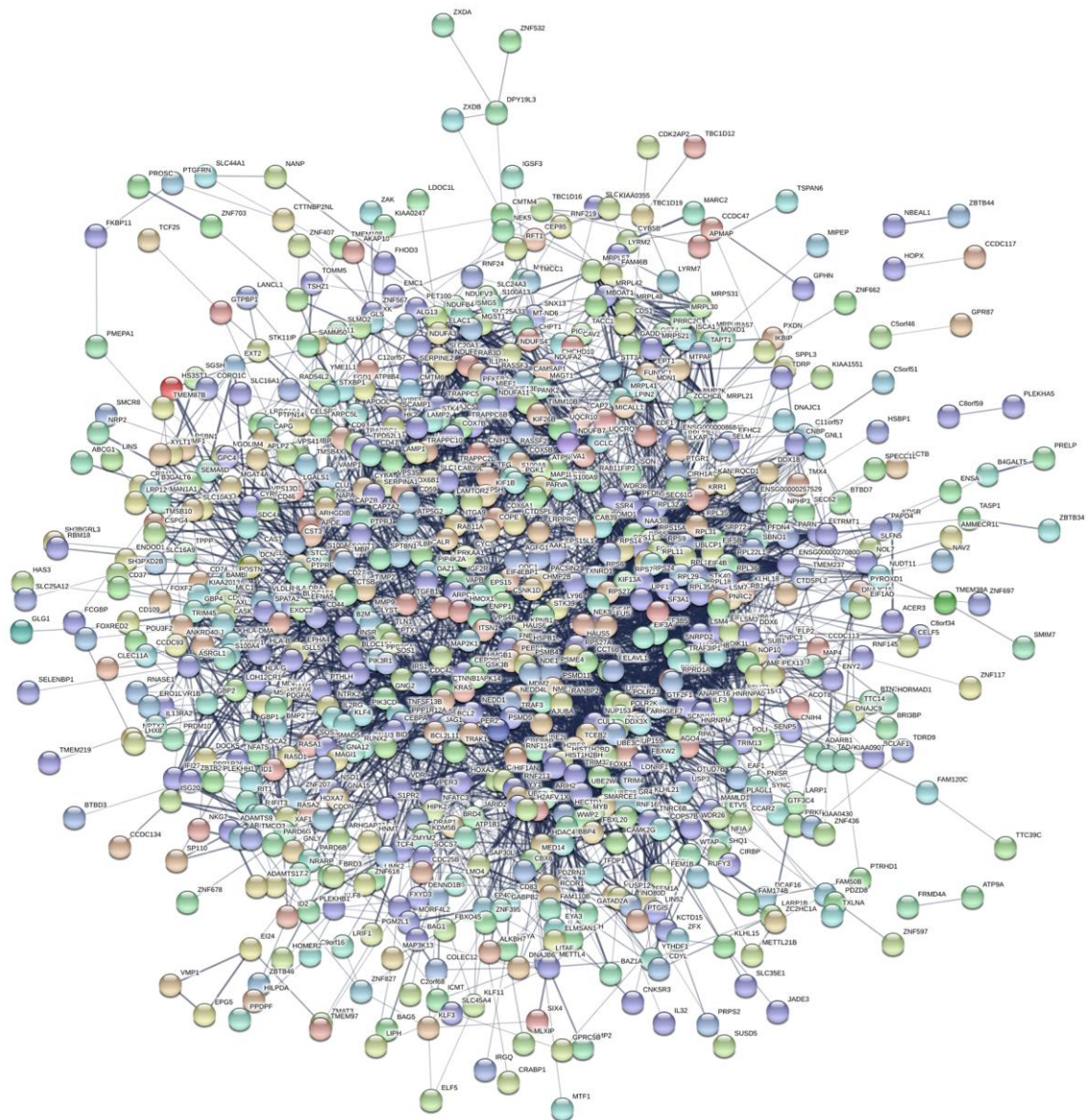

Supplement: Supplementary Figure S1 [file BSR-2020-0869_supp.pdf]
